# Second Primary Malignancies in Patients with Colorectal Cancer: The Frequency, Types, and Timeline

**DOI:** 10.3390/jcm15031053

**Published:** 2026-01-28

**Authors:** Zlata Lerner, Sarah Weissmann, Heba Abu-Kaf, Ali Abu-Juma, Barah Abu Gnim, Naim Abu-Freha

**Affiliations:** 1 The Institute of Gastroenterology and Hepatology, Soroka University Medical Center, P.O. Box 151, Beer-Sheva 84101, Israel; zlata.31@gmail.com (Z.L.); ak.heba@gmail.com (H.A.-K.); ali.abu.jumaa91@gmail.com (A.A.-J.); 2 Faculty of Health Sciences, Ben-Gurion University of the Negev, Beer-Sheva 84105, Israel; abugnimb@post.bgu.ac.il; 3 Medical School for International Health, Ben-Gurion University of the Negev, Beer-Sheva 84105, Israel; sarahgrzebinski@gmail.com

**Keywords:** colorectal cancer, second primary malignancy, Lynch syndrome, breast cancer, prostate cancer

## Abstract

**Background/Objectives**: Colorectal cancer (CRC) is the third most common cancer worldwide. Accordingly, we aimed to investigate the frequency, types, and timelines of other cancers in patients diagnosed with CRC. **Methods**: Patients diagnosed with CRC between 1999 and 2023 were retrospectively included. We collected data on the demographics and diagnosis of any additional cancer, either prior to or following the CRC diagnosis. Data were retrieved from the Clalit database using the MDClone platform. **Results**: A total of 65,774 CRC patients were identified between 1999 and 2023. A second primary malignancy (SPM) was diagnosed in 13,679 patients (21%). Being female, smoking, and having chronic obstructive pulmonary disease were more frequent among patients with a SPM. Breast, prostate, and lung cancers were the most prevalent SPMs. The risk of developing SPM was highest during the first three years after CRC diagnosis, especially with respect to lung cancer, whereas the majority of breast and prostate cancers were diagnosed during the three years before CRC; the cumulative standardized incidence ratio was calculated to be 4.1 for any cancer. **Conclusions**: CRC patients have an increased second primary cancer. Patients diagnosed with CRC should be encouraged to undergo screening for other cancers, and those with Lynch-related cancers should be investigated for the syndrome. Patients with a diagnosis of breast or prostate cancer should be considered for colonoscopy screening.

## 1. Introduction

Colorectal cancer (CRC) is the third most common cancer and the second leading cause of cancer-related deaths globally [1,2]. It is estimated that there were 1.8 million new cases and about 881,000 deaths worldwide in 2018 [1]. There is wide geographical variation in CRC incidence and mortality, with variation in incidence for different ages, sexes, and racial groups. This variation could contribute to exposure to the different risk factors and genetic susceptibility. At diagnosis, the median age of patients with colon cancer is 66 and 69 in men and women, respectively [3], but it is lower for rectal cancer (62 and 63, respectively) [3]. CRC screening is recommended for the 50–75-year-old population in most countries to detect precancerous lesions and CRC in its early stages. However, implementation of CRC-screening programs varies geographically [4], and the compliance rate ranges from 16% to 93% in the first round of screening, depending on the screening test used [4]. Despite the recommendations for CRC screening in the guidelines, the implementation of screening varies significantly among countries in different regions. There are differences in the screening methods, including with respect to non-invasive stool testing, imaging techniques, and endoscopy, and new screening modalities, with implementation of organized or opportunistic screening programs. Improvements in early detection and multimodal therapy have steadily increased survival, expanding the population of CRC survivors who live long enough to develop new malignancies, including the second primary malignancy (SPM). In general, synchronous SPMs are those diagnosed around the time of diagnosis of the index tumor (within 2–6 months), while metachronous SPMs are not diagnosed around the same time the index tumor is diagnosed.

Previous studies showed that CRC patients are at an increased risk for SPMs; about 20% will develop an SPM [5,6]. This issue is of increasing importance due to the rising incidence of CRC in younger age groups and the increased life expectancy of CRC patients.

The 3-year, 5-year, and 10-year cumulative risks of developing an SPM were found to be 3.9%, 5.9%, and 10.0%, with a median survival time after the diagnosis of the SPM of less than 4 years [7]. Being male and having survived colon cancer, being older, being afflicted with a well-differentiated tumor, having localized disease, and having undergone surgery were found to be risk factors for SPMs [7]. The frequency of SPMs depends on population, follow-up length, age, and study methodology.

The risk of developing SPMs can be attributed to several factors, including shared risk factors for both cancers, the index tumor, and the second primary tumor (such as smoking, obesity, and a family history of cancer); genetic predisposition; and the potential effects of cancer treatments on the body’s immune system [8,9].

In addition to metachronous CRC, an increased risk of different malignancies has been reported, including prostate, breast, ovarian, stomach, urinary bladder, and other cancers [10].

In this retrospective study, we aimed to investigate SPMs among CRC patients in a national big-data setting with a large patient cohort and a long-term follow-up and describe the main risk factors and the most frequent SPMs in CRC patients and the timeline of SPMs’ occurrence in relation to the CRC diagnosis.

## 2. Materials and Methods

### 2.1. Population and Study Setting

Patients aged 18 and older diagnosed with CRC (extracted from the diagnosis list in the community clinic or hospitals) were enrolled in the analysis. The following ICD-9 codes were included: 153 (excluding 153.5, appendix vermiformis) and 154 (excluding 154.2 and 154.3 and anal cancer). The data were extracted from Clalit Health Services (CHS) using Clalit’s data-sharing platform powered by MDClone (https://www.mdclone.com, Beer-Sheva, Israel). CHS is the largest health maintenance organization (HMO) in Israel, containing data from about 6 million people. The MdClone platform includes computerized data on the patients and data from community clinics, emergency departments, and hospitals. Data is available for every patient and includes diagnoses; events such as hospitalizations, emergency department visits, and surgeries; laboratory results; and medication prescriptions. Any new diagnoses, hospitalizations, or medical updates are recorded as new events. Longitudinal data organization makes it possible to explore events on a linear timeline. Anonymous patient data can be extracted in relation to other recorded events. The MdClone platform enables exploration of large cohorts, long-term data collection, and a holistic view of the patients from birth to death.

SPMs were characterized as diagnoses of malignancy before or after the CRC diagnosis, excluding second diagnoses of CRC. Cancer diagnoses undergo routine validation through a centralized registry that ensures that any patient with a confirmed malignancy is documented in the medical record. Nevertheless, our study relied on ICD-9 diagnostic codes rather than direct review of pathology reports. As with any code-based dataset, there remains a possibility of misclassification.

### 2.2. Data Collection

Demographics, comorbidities, malignancies, and mortality rates pertaining to patients with and without SPMs were retrospectively collected according to the ICD-9 diagnosis codes. Data regarding comorbidities (Diabetes Mellitus (DM), obesity, dyslipidemia, hypertension, chronic ischemic heart disease (CIHD), cerebrovascular accident (CVA), chronic obstructive lung diseases (COPDs), and renal failure) were collected. In addition, the frequency of common malignancies was collected (lung, prostate, breast, esophageal, stomach, pancreatic, kidney, urinary bladder, uterine, and ovarian cancers; Hodgkin’s and non-Hodgkin’s lymphoma; and thyroid malignancy).

### 2.3. Statistical Analysis

Data are presented as means ± standard deviations (SDs) for continuous variables and as percentages (%) of the total for categorical variables. Univariate analyses were performed using the Mann–Whitney test for continuous variables and Fisher’s exact and chi-squared tests for categorical variables. All statistical analyses were performed using IBM SPSS version 28 (Chicago, IL, USA). *p*-values less than 0.05 were considered statistically significant. The cumulative standardized incidence ratio (SIR) was calculated for each site-specific second primary malignancy among CRC patients in comparison to the observed cases in the reference general population (aged 18 years and older) in the database, which consisted of individuals aged ≥18 years (n = 4,811,886, females = 2,448,629, males = 2,363,357).

The study protocol was approved by the Institutional Helsinki Committee, under approval number 87-22-SOR-SOR, and it was carried out according to the principles of the Declaration of Helsinki.

## 3. Results

### 3.1. Study Population

We obtained data from 65,774 patients with CRC over a span of 24 years (1999–2023). SPMs were diagnosed in 13,679 cases (21%), before or after the index CRC diagnosis. The baseline characteristics of CRC patients with and without a second primary malignancy are shown in Table 1.

Patients with SPM were older (72 ± 12 vs. 69.7 ± 14 years, *p* < 0.001). There was a higher proportion of females (54% vs. 48%, *p* < 0.001), a lower proportion of Arab patients (6% vs. 9.1%, *p* < 0.001), and a lower proportion of a family history of CRC (9.2% vs. 10.3%, *p* < 0.001) among patients with an SPM. The patients with SPMs were more likely to be smokers or diagnosed with COPD (33.1% vs. 29.8%, *p* < 0.001, and 14.9% vs. 9.6%, *p* < 0.001).

Patients without SPMs were more likely to suffer from metabolic comorbidities such as CIHD and DM, with frequency rates of 15.1% vs. 12.9% (*p* < 0.001) and 30.9% vs. 29% (*p* < 0.001), respectively.

All-cause mortality rates were higher among patients with SPMs (70% vs. 58%). However, the survival period from the time of CRC diagnosis was longer (5.4 years vs. 4.5 years, *p* < 0.001).

### 3.2. Second Primary Malignancies

The types of SPMs, the timing of their diagnosis, and the cumulative standardized scores for SPMs (total SPMs and SPMs after CRC) are shown in Table 2.

A total of 21% of patients with CRC were diagnosed with SPMs (not including a second primary CRC). Around 11% of SPMs were diagnosed after the diagnosis of the index CRC and 11% before CRC. The three most common SPMs in our cohort were breast (8%, SIR = 3.3), prostate (4.8%, SIR = 4.5), and lung (3%, SIR = 3.7) cancers. Lung cancer was diagnosed more frequently after the CRC diagnosis (2.1% vs. 1.1%), whereas the majority of breast and prostate cancers preceded the CRC diagnosis (4.6% vs.3.3% and 5.2% vs. 4.5%, respectively).

Other less frequent malignancies such as stomach, kidney, uterus, ovarian, bladder, and pancreas cancer, which are known to be part of the group of Lynch syndrome cancers, were also diagnosed: stomach—1.1% before CRC and 1% after CRC; uterus—1.2% before CRC and 1.1% after CRC; ovarian—1.3% before CRC and 0.9% after CRC; bladder malignancy—1.5% before CRC and 1.4% after CRC; and pancreatic—0.3% before CRC and 0.7% after CRC. The cumulative SIRs are presented in Table 2; the SIRs range from 1.7 to 10.8 across the site-specific cancers investigated. After considering the SPMs cases diagnosed after CRC, the cumulative SIRs are higher in all cancer types investigated in our study (except lymphoma).

### 3.3. Second Primary Malignancies and Age Groups

Table 3 shows the number and percentages of SPMs among females and males across various age groups; the age groups were subdivided according to the patients’ ages when diagnosed with CRC.

For females, the distribution of SPMs is relatively uniform between the ages of 31 and 70 years, but it is lower for younger and older patients. This pattern likely reflects the shorter life expectancy of patients diagnosed with CRC at older ages. In females, breast cancer is the most common SPM, with the highest proportion corresponding to the 61–70 age group. A total of 8.7% of the patients in this age group were diagnosed with breast cancer, while there was a relatively lower proportion for the other age groups between the ages of 31 to 70. Kidney and urinary bladder malignancies become more common with age, peaking in the cohort older than 61, with frequencies of 1.4% for kidney malignancy and 1.4% for urinary bladder malignancy. Uterine and ovarian malignancies are more common in middle-aged groups, with ovarian malignancy peaking at 3.6% in the 41–50-year-old group. The frequency decreases with age, with values of 2.9% in the 51–60 age group, 2.4% in the 61–70 age group, 1.6% in the ≥71 age group. Uterine malignancy peaks at 2.7% in the 61–70-year-old group, with a frequency rate ranging from 0.3% to 2.6% in the other age groups.

In males, there is a linear increase in the percentage of SPMs with older age upon CRC diagnosis. Lung cancer is prevalent across all age groups, with the highest percentage (4.5%) being in the 61–70-year-old age group, while 1.2% of the age group ranging between 31 and 40 years was diagnosed with lung cancer. Prostate cancer is increasingly common with age, starting at 0.7% in the 31–40-year-old group and rising to 12.8% in the ≥71-year-old group. The frequency of kidney malignancy increases with age, affecting around 2.8% of patients 71 years and older; in the same vein, there is an increase in the proportion of urinary bladder malignancy with age among males, affecting 6% of patients aged 71 and older. The frequency rates of other, less common cancers such as stomach and pancreatic cancer, lymphoma, and non-Hodgkin’s lymphoma also increase with age. The trend regarding lung, prostate, kidney, and urinary bladder cancers demonstrates a gradual rise across successive age categories, reflecting the cumulative burden of SPMs in older patients. While lung cancer was consistently observed in all strata, prostate malignancy showed the most pronounced age-related escalation. Similarly, kidney and urinary bladder cancers, although less frequent overall, revealed a steady upward pattern beyond the age of 50.

### 3.4. Lynch-Related Cancers

A sub-analysis of SPMs revealed that 16.8% of all cases were suspected to be related to Lynch syndrome, based on an age at CRC diagnosis of 50 years or younger (9.1%), with 5974 patients diagnosed at or before age 50. Regarding second primary Lynch-related cancers, 5093 (8.5%) of cases were found to be afflicted with a second primary malignancy before (2633, 4.3%) or after (2544, 4.3%) developing CRC. Importantly, there was no significant difference between the diagnosis of Lynch-related SPMs before and after CRC (both 4.3%). The results are summarized in Table 4.

### 3.5. Timeline of SPM

**Figure 1:**
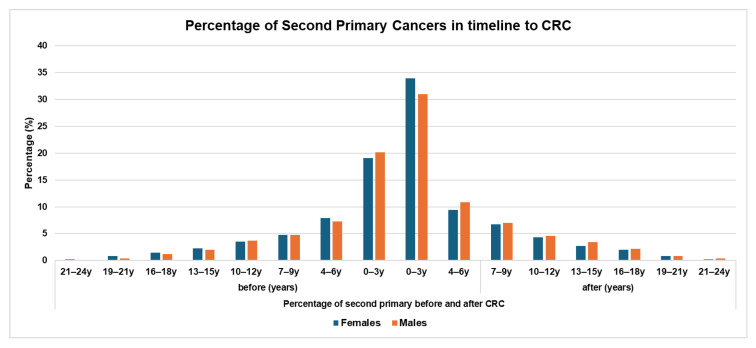
Percentage of second primary cancers in the timeline of CRC.

The timing of SPMs in relation to CRC diagnosis is shown in Figure 1. SPMs are more frequently diagnosed within 0–3 years before and after the index CRC in both females and males. As the time since CRC diagnosis increases, there is a gradual decline in the percentage of new SPM cases. The distribution curve demonstrates a clear concentration of SPM diagnoses around the peri-diagnostic period, followed by a steady reduction over subsequent years. This pattern is consistent across sexes, with the highest clustering observed immediately surrounding the CRC diagnosis. Ten years after the initial CRC, the proportion of newly diagnosed SPMs becomes notably lower.

## 4. Discussion

Our study provides important insights regarding SPMs in CRC patients. We found that 21% of CRC patients in our cohort had SPM, which could have developed before or after CRC. Only a few studies have evaluated similar cohorts, and, to date, there are no special recommendations for CRC patients with SPMs.

The prevalence of SPMs found in our study was similar to that reported in other studies. One study, which included 1174 patients, found that SPMs were reported in 20% of patients [11], while another study, which included 65,648 CRC patients, found SPMs in 6% (3810 cases) [12]. This difference in reported prevalence may be related to the number of patients included, the study cohort, and/or the follow-up period. In this study, we included SPMs that developed before and after the index CRC, and the prevalence of SPMs could be attributed to shared risk factors, genetic predisposition, and the long-term effects of cancer treatments. Importantly, SPM occurrence is likely multifactorial and could be due to shared risk factors such as smoking, alcohol consumption, obesity, and dietary factors, which have been shown to predispose individuals to multiple primary cancers [8,9]. Genetic predisposition, including with respect to hereditary cancer syndromes, is also strongly linked with an increased risk of SPMs in CRC patients. However, the frequency of these risk factors varies in different populations.

Breast, prostate, and lung cancer were the most common SPMs in our study. These cancers are common in the general population. This finding emphasizes the importance of screening CRC patients for other cancers. However, the cumulative SIRs are increased in CRC patients (for all site-specific cancers investigated in our study) compared to the general population of our database. Breast and prostate cancer were also among the most common types of SPMs in other studies [11]. In a meta-analysis that included 42 publications, CRC survivors were found to be more likely to develop secondary neoplasms of the prostate (with a pooled standardized incidence rate (SIR) of 1.22), breast (female) (SIR 1.18), ovary (SIR 1.95), stomach (1.12), urinary bladder (SIR 1.21), kidney (SIR 1.66), thyroid (SIR 1.98), bone, and soft tissue (SIR 1.33) [10]. Our study revealed that 8% of the female patients had female breast cancer, with 4.6% of the cancers developing before the CRC diagnosis. Furthermore, 4.8% and 3.7%% of the male CRC patients were diagnosed with prostate cancer and lung cancer, respectively. In addition, in previous studies, an increased risk of CRC was found among breast and prostate cancer patients [13,14,15], with an SIR of 1.41 for CRC among breast cancer survivors [13]. Among prostate cancer patients, CRC was the third most common cancer, with 12.2% of the SPMs developing after lung and urinary bladder cancers [14]. In another study that included 76,614 prostate cancer patients, 8659 (11.3%) were diagnosed with a SPM, and a relative risk of 1.32 was found for the diagnosis of CRC.

Regarding sex and age, our study demonstrates that the frequency rates of SPMs increase with age for most cancers, particularly among patients diagnosed with CRC at an age of over 50 years. With respect to the sex of CRC patients, the most common SPM diagnosed was breast cancer among females and prostate cancer among males, while lung cancer was common among both females and males. Other cancers were less common among both sexes.

Regarding risk factors and comorbidities, a high percentage of patients with SPMs were smokers. Smoking is a known risk factor for different cancers, increasing the risk not only for CRC but also for other cancers such as lung cancer [16].

Multiple mechanisms are involved in the carcinogenic potential of tobacco smoke, including the induction of DNA adducts, impairment of DNA repair pathways, and promotion of systemic inflammation, all of which contribute to multi-organ tumorigenesis [17].

Patients without SPMs were less likely to suffer from metabolic comorbidities such as CIHD and DM. Patients with chronic disease may receive medical attention, attend follow-ups, and receive encouragement for a healthy lifestyle and screening more frequently. Moreover, the detection of precancerous lesions and early cancers, potentially preventing progression to clinically apparent SPMs, could be increased in this regard as a result of the surveillance effect [18]. Furthermore, patients with significant cardiovascular or metabolic disease may have a shorter life expectancy, which could reduce their likelihood of living long enough to develop or be diagnosed with SPMs. The prevalence of comorbidities varies significantly in different studies and populations [18]. In our study, some of the comorbidities were common; about 50% of the patients had hypertension, about 29% had DM, 24% had obesity, and 13% had CIHD. Despite the statistical significance of the differences regarding some of the comorbidities among those who developed SPMs relative to those who did not have SPMs, the differences are small and of limited clinical significance. Metabolic comorbidities are also associated with an increased risk of cancer. Diabetes and obesity contribute to pro-inflammatory, hyperinsulinemic, and hyperglycemic states, which may promote tumor growth in certain organs [19]. However, in general, changes in hormone profiles, growth factors, steroids, and specific treatments such as anti-diabetic treatments could play a protective role and have an antineoplastic effect [18]. Overall, on the one hand, the direct carcinogenic effect of the comorbidities in terms of inflammatory and metabolic pathways influences the incidence of SPMs, while on the other hand, the increased medical surveillance and limited survival time have protective effects. The balance between carcinogenic and protective effects seems to be dynamic and context-dependent, and this aspect likely determines the observed outcomes in the different studies and populations. In our study, the all-cause mortality was found to be higher among those with an SPM, reaching 70.1% during the follow-up period relative to 58.7% for those without an SPM. However, because information on the specific cause of death and cancer-related death was not available, it is not possible to determine whether this excess mortality was directly attributable to the SPM itself. This limitation underscores the importance of incorporating cause-specific mortality data in future research to better clarify the impact of SPMs on survival outcomes.

One of our important findings is the timing of the SPMs in relation to CRC. Most SPMs were diagnosed three years before or after the original CRC diagnosis. This finding is in agreement with previous studies [10,20] and may be attributable to several factors, including increased medical surveillance, more screening, more frequent follow-ups, and increased performance of diagnostic tests such as imaging, resulting in early detection of other cancers. Moreover, during the diagnostic period, CRC patients undergo extensive staging procedures, including imaging, which can uncover incidental precancerous lesions or neoplasms, which might otherwise remain undetected. In addition, the psychological impact of a cancer diagnosis could prompt patients to seek more medical evaluation and adhere to the recommended screening programs, resulting in the detection of different cancers. These findings highlight the importance of screening CRC patients for other cancers around the time of diagnosis and allow us to recommend screening patients diagnosed with breast or prostate cancer for CRC.

Another important insight of our study is the risk for Lynch syndrome and the need for further investigation according to the revised Bethesda criteria [21]. Lynch syndrome is the most common hereditary predisposition for CRC, which is caused by germline mutations in DNA mismatch repair (MMR) genes (MLH1, MSH2, MSH6, and PMS2) or deletion of EPCAM, leading to microsatellite instability and accelerated carcinogenesis. The syndrome is characterized by CRC at a young age and a risk for synchronous and metachronous second primary cancers. Based on the age at which the individuals were diagnosed with CRC or SPMs of Lynch-related cancers, approximately 16% of the patients in our cohort should be further screened for Lynch syndrome. This high frequency can be attributed to the long study period (more than 20 years) and inclusion of both of the following two criteria: diagnosis with CRC at an age younger than 50 and a second Lynch-related cancer before or after the index CRC. In a recently published meta-analysis that included 51 studies, an overall pooled prevalence of pathological germline variants of 2.2% was found for CRC patients, ranging from 0.4% to 21.2% [22]. This frequency is much higher than that noted in the published literature. Unfortunately, we were unable to confirm the diagnosis of Lynch syndrome via genetic testing. However, as recommended by several associations, universal screening of CRC and endometrial cancer patients could be the first step in the investigation and be helpful for Lynch syndrome diagnosis [23]. As 16% of the CRC patients in our cohort needed to be screened for Lynch syndrome, it is important for clinicians, including oncologists, gastroenterologists, and geneticists, to recognize this condition and refer these patients for further investigation for Lynch syndrome. Identifying Lynch syndrome has direct implications not only for index patients in terms of tailored surveillance but also for undiagnosed family members who may harbor the same pathogenic variants. The relatively high proportion of patients for whom Lynch syndrome evaluation was warranted in our cohort underscores the need for multidisciplinary collaboration (among gastroenterologists, oncologists, pathologists, and genetic counselors) in order to optimize both patient care and familial cancer prevention strategies.

In summary, the diagnosis of an SPM among CRC patients is of unique importance in clinical practice. In this study, we highlighted several important insights, including (1) a frequency rate of about 21% of SPMs; (2) the fact that certain types of cancer are more common, such as breast cancer, prostate cancer, and lung cancer, with a frequency that increases with age and for a specific sex; (3) the observation that risk factors and comorbidities seem to play an important role in the carcinogenic or protective effect regarding SPMs; (4) an increased prevalence of SPMs within the first three years of CRC diagnosis; and (5) the need for further investigation concerning Lynch syndrome in about 16% of our cohort. To reduce the risk of developing an SPM, patients with CRC should adhere to a healthy lifestyle, avoid risk factors such as smoking, and undergo regular cancer screening so that any new cancers can be detected early. Additionally, CRC patients should be aware of any potential symptoms of new cancers and promptly report any changes in their health to their healthcare providers.

### Clinical Implications and Future Directions

There are several important clinical implications for clinical practice, including the need to increase clinicians’ awareness of the importance of screening CRC survivors for other cancers. General practitioners, oncologists, and gastroenterologists need to inform CRC survivors about their risk of developing additional primary cancers and encourage them to undergo screening for other cancers and attend further follow-ups. Moreover, patients diagnosed with other cancers, such as prostate or breast cancer, should be referred to for CRC screening.

The three-year window around CRC diagnosis has practical clinical implications: it represents a critical period during which heightened vigilance for SPMs is warranted. The diagnosis of SPMs around the index time of CRC diagnosis has been reported in different studies, emphasizing and highlighting the importance of screening during this period. Moreover, these findings highlight the importance of completing diagnostic work-ups and surveillance, which are helpful for diagnosing metachronous SPMs, and performing screening for other cancers in this population.

Further studies, including prospective studies, need to be conducted in order to prospectively identify subgroups of CRC patients with specific risk factors, including the type of CRC treatment received. Moreover, developing predictive models for SPMs could be an important tool and helpful for clinicians in their daily practices.

The main strength of this study lies in its long-term scope and use of big data, including real-world data, allowing it to generate meaningful population-level insights and increasing the generalizability of the findings. This broad dataset increases statistical power and allows the detection of trends over time. Importantly, the design of this study differs from other studies in terms of the timing of the SPMs, as we included SPMs from the entire study period, whether they occurred before or after the CRC, rather than only those that developed after the CRS, as in most studies.

However, several limitations should be acknowledged, including this study’s retrospective nature and the lack of specific information such as the stage, histology, localization, and genetic testing of the CRC, making it unfeasible to perform causal or mechanistic analyses. In addition, no data were available regarding oncological treatments or the cause of death or CRC-related mortality. Another limitation is that cancer diagnoses were based solely on ICD-9 codes without direct pathology confirmation, potentially resulting in occasional diagnostic misclassification. Moreover, this study covers a 24-year period, and diagnostic technologies, screening recommendations, and clinical practices for both CRC and other cancers have changed considerably over time. This fact should be taken into account when interpreting the findings.

## 5. Conclusions

Twenty-one percent of patients diagnosed with CRC have also been diagnosed with a second primary malignancy, particularly within the three years before and after the initial diagnosis. This pattern is likely influenced by diagnostic and surveillance practices rather than an inherent short-term biological predisposition. It is crucial to encourage CRC patients to remain in follow-ups and undergo screening for other cancers. Patients diagnosed with breast or prostate cancer should be considered for colonoscopy screening. Healthcare providers should ensure that appropriate, guideline-based screening for other cancers is maintained regarding this population; be aware of the risk of Lynch syndrome (according to known clinical criteria, particularly age and metachronous cancer); and pursue further investigations when appropriate.

## Figures and Tables

**Table 1:** Baseline characteristics of patient groups, with and without a second primary malignancy.

	Second Primary Anytime n = 13,679 (%)	No Second Primary n = 52,095 (%)	*p*-Value
Age at CRC diagnosis	72,1 ± 11.6	69.7 ± 14	<0.001
Gender, female	7382 (54)	25,108 (48)	<0.001
Smoking	2961 (33.1)	9846 (29.8)	<0.001
Ethnicity: Arab	825 (6)	4719 (9.1)	<0.001
FHCRC	1261 (9.2)	5388 (10.3)	<0.001
BMI	28.3 ± 5.8	28.3 ± 5.9	0.984
Comorbidities			
CIHD	2068 (15.1)	6728 (12.9)	<0.001
COPD	2037 (14.9)	4981 (9.6)	<0.001
Asthma	777 (5.7)	2925 (5.6)	0.767
CKD	1831 (13.4)	5423 (10.4)	<0.001
HTN	8152 (59.6)	27,932 (53.6)	<0.001
DM	4227 (30.9)	15,084 (29)	<0.001
CVA	287 (2.1)	1077 (2.1)	0.822
Obesity	3256 (23.8)	12,460 (23.9)	0.779
Death	9583 (70.1)	30,597 (58.7)	<0.001
Survival, mean ± SD	5.4 ± 5.4	4.5 ± 5	<0.001

FHCRC = family history of colorectal cancer, BMI = Body Mass Index, CIHD = Chronic Ischemic Heart Disease, COPD = chronic obstructive pulmonary disease, CKD = Chronic Kidney Disease, HTN = Hypertension, DM = Diabetes Mellitus, and CVA = Cerebrovascular Accident.

**Table 2:** Second primary cancers before and after CRC diagnosis.

	Cancers Before CRC (%) *	Cancers After CRC (%) *	Total	Cumulative SIR	Cumulative SIR After CRC
Lung cancer	703 (1.1)	1370 (2.1)	2073 (3)	3.7	2.4
Prostate cancer (males)	1688 (5.2)	1475 (4.5)	3163 (4.8)	4.5	2.1
Stomach malignancy	705 (1.1)	674 (1)	1379 (2.1)	9.3	4.4
Breast malignancy (females)	1534 (4.6)	1113 (3.3)	2647 (8)	3.3	1.4
Pancreatic malignancy	196 (0.3)	473 (0.7)	669 (1)	4	2.8
Kidney malignancy	592 (0.9)	656 (1)	1248 (1.9)	4.8	2.5
Uterus malignancy (females)	387 (1.2)	367 (1.1)	754 (2.3)	8	3.9
Ovarian malignancy (females)	420 (1.3)	289 (0.9)	709 (2.1)	7.7	3.1
Non-Hodgkin’s lymphoma	365 (0.6)	337 (0.5)	702 (1.1)	3.6	1.7
Lymphoma	65 (0.1)	54 (0.1)	119 (0.2)	1.7	0.7
Esophageal malignancy	98 (0.1)	183 (0.3)	281 (0.4)	5.8	3.7
Thyroid malignancy	142 (0.2)	190 (0.3)	332 (0.5)	2.5	1.4
Urinary bladder malignancy	965 (1.5)	913 (1.4)	1878 (2.9)	4.8	2.3
Any cancer patients	7227 (11)	7201 (10.9)	13,679 (20.8)	4.1	2.2

* The proportions are from the entire cohort of 65,774 CRC patients or sex specific proportion (females = 33,284). SIR = Standardized Incidence ratio.

**Table 3:** Cancer frequency according to the age of CRC diagnosis.

CRC Age Females n = 33,284	≤30 Years n = 299	31–40 Years n = 734	41–50 Years n = 2101	51–60 Years n = 4690	61–70 Years n = 7595	≥71 Years n = 17,865
Lung cancer	3 (1)	5 (0.7)	21 (1)	115 (2.5)	214 (2.8)	415 (2.3)
Stomach malignancy	3 (1)	10 (1.4)	32 (1.5)	80 (1.7)	125 (1.6)	291 (1.6)
Breast malignancy	5 (1.7)	30 (4.1)	86 (4.1)	344 (7.3)	658 (8.7)	1522 (8.5)
Pancreatic malignancy	0	3 (0.4)	10 (0.5)	27 (0.6)	87 (1.1)	178 (1)
Kidney malignancy	0	1 (0.1)	13 (0.6)	35 (0.7)	102 (1.3)	257 (1.4)
Uterus malignancy	1 (0.3)	9 (1.2)	47 (2.2)	121 (2.6)	207 (2.7)	368 (2.1)
Ovarian malignancy	5 (1.7)	22 (3)	76 (3.6)	137 (2.9)	181 (2.4)	288 (1.6)
Non-Hodgkin’s lymphoma	4 (1.3)	3 (0.4)	9 (0.4)	23 (0.5)	87 (1.1)	184 (1)
Lymphoma	0	3 (0.4)	3 (0.1)	5 (0.1)	16 (0.2)	22 (0.1)
Thyroid malignancy	7 (2.3)	2 (0.3)	16 (0.8)	46 (1)	59 (0.8)	96 (0.5)
Urinary bladder malignancy	2 (0.7)	3 (0.4)	17 (0.8)	34 (0.7)	106 (1.4)	232 (1.3)
Any cancer	27 (9)	83 (11.3)	291 (13.9)	843 (18)	1644 (21.6)	3049 (19.1)
**Males** **n = 32,490**	n = 282	n = 687	n = 1871	n = 4519	n = 8344	n = 16,787
Lung cancer	0	8 (1.2)	38 (2)	164 (3.6)	378 (4.5)	625 (3.7)
Prostate cancer	0	5 (0.7)	35 (1.9)	210 (4.6)	733 (8.8)	2153 (12.8)
Stomach malignancy	2 (0.7)	9 (1.3)	44 (2.4)	98 (2.2)	190 (2.3)	495 (2.9)
Pancreatic malignancy	1 (0.4)	6 (0.9)	17 (0.9)	53 (1.2)	120 (1.4)	167 (1)
Kidney malignancy	1 (0.4)	1 (0.4)	25 (1.3)	117 (2.6)	219 (2.6)	476 (2.8)
Non-Hodgkin’s lymphoma	4 (1.4)	6 (0.9)	11 (0.6)	48 (1.1)	105 (1.3)	218 (1.3)
Lymphoma	0	5 (0.7)	6 (0.3)	17 (0.4)	12 (0.1)	30 (0.2)
Thyroid malignancy	0	3 (0.4)	5 (0.3)	20 (0.4)	29 (0.3)	49 (0.3)
Urinary bladder malignancy	0	5 (0.7)	37 (2)	94 (2.1)	362 (4.3)	986 (5.9)
Any cancer	8 (2.8)	47 (6.8)	197 (10.5)	729 (16.1)	1907 (22.9)	4494 (26.8)

**Table 4:** Age and malignancies related to Lynch syndrome.

65,774 CRC Cases				
Age 50 Years or Younger	Lynch-Related Cancer Before CRC	Lynch-Related Cancer After CRC	Lynch-Related Cancer Anytime	Total Cases Susp. to be Lynch Syndrome
5974 (9.1)	2633 (4.3)	2544 (4.3)	5093 (8.5%)	11,067 (16.8%)

## Data Availability

No additional data will be available.

## Abbreviations

The following abbreviations are used in this manuscript:

CRC	Colorectal cancer
SPM	Second primary malignancy
FHCRC	Family history of colorectal cancer
BMI	Body mass index
CIHD	Chronic ischemic heart disease
COPD	Chronic obstructive pulmonary disease
CKD	Chronic kidney disease
HTN	Hypertension
DM	Diabetes mellitus
CVA	Cerebral vascular accident
